# High-Frequency Oscillatory Ventilation Combined with Volume Guarantee in a Neonatal Animal Model of Respiratory Distress Syndrome

**DOI:** 10.1155/2013/593915

**Published:** 2013-07-18

**Authors:** Manuel Sánchez Luna, Martín Santos González, Francisco Tendillo Cortijo

**Affiliations:** ^1^Neonatology Division, Instituto de Investigación Sanitaria Gregorio Marañón, Hospital General Universitario “Gregorio Marañón”, C/ Dr. Esquerdo 46, 28007 Madrid, Spain; ^2^Medical and Surgical Research Unit, Instituto de Investigación Sanitaria Puerta de Hierro Majadahonda, Hospital Universitario Puerta de Hierro Majadahonda, C/ Manuel de Falla 1, 28222 Madrid, Spain

## Abstract

*Objective*. To assess volume guarantee (VG) ventilation combined with high-frequency oscillatory ventilation (HFOV) strategy on PaCO_2_ regulation in an experimental model of neonatal distress syndrome. *Methods*. Six 2-day-old piglets weighing 2.57 ± 0.26 kg were used for this interventional experimental study. Animals were ventilated during physiologic lung conditions and after depletion of lung surfactant by bronchoalveolar lavage (BAL). The effect of HFOV combined with VG on PaCO_2_ was evaluated at different high-frequency expired tidal volume (VThf) at constant frequency (*f*_*R*_) and mean airway pressure (mPaw). Fluctuations of the pressure (ΔPhf) around the mPaw and PaCO_2_ were analyzed before and after lung surfactant depletion. *Results*. PaCO_2_ levels were inversely proportional to VThf. In the physiological lung condition, an increase in VThf caused a significant decrease in PaCO_2_ and an increase in ΔPhf. After BAL, PaCO_2_ did not change as compared with pre-BAL situation as the VThf remained constant by the ventilator. *Conclusions*. In this animal model, using HFOV combined with VG, changes in the VThf settings induced significant modifications in PaCO_2_. After changing the lung condition by depletion of surfactant, PaCO_2_ remained unchanged, as the VThf setting was maintained constant by modifications in the ΔPhf done by the ventilator.

## 1. Introduction

High-frequency oscillatory ventilation (HFOV) is characterized by an effective gas exchange using tidal volumes equal to or less than the dead space volume [1] at supraphysiological frequencies. Carbon dioxide (CO_2_) removal is mostly related to the tidal volume generated during high-frequency ventilation [2–5], and this high-frequency expired tidal volume (VThf) is known to be close to the airway dead space [6]. Also, the frequency (*f*_*R*_) has a role in CO_2_ removal [7] and has an independent effect on the distribution of the gas within the airways [8]. During HFOV, several mechanisms of gas exchange have been described, the combination of which is responsible for its ventilatory efficiency [9]. As HFOV uses a tidal volume lower or equal to the anatomical dead space, it has been argued that the incidence of bronchopulmonary dysplasia (BPD) can be reduced [10]. Although other potential mechanisms have been described by which HFOV causes less ventilator-induced lung damage [11], the prevalence of BPD in preterm infants treated with HFOV is similar to those undergoing conventional ventilation [12].

VThf is crucial for CO_2_ elimination and has a larger impact on CO_2_ removal during HFOV in comparison to tidal volume during conventional mechanical ventilation. VThf is mostly generated by fluctuations of the pressure (ΔPhf) around the mean airway pressure (mPaw) and to the frequency *f*_*R*_ [13], but also the amount of VThf can be related to the endotracheal tube characteristics and compliance of the tubing as well as performance of the ventilator and lung conditions. Accordingly, large variations of VThf are possible during HFOV, which in turn may cause unexpected variations of CO_2_ removal [14].

VThf delivery depends on the ventilator characteristics, and although VThf monitoring is useful in daily practice, most of the ventilators providing HFOV do not display or measure the VThf [14–19]. However, in some new HFOV ventilators it is possible to adjust directly the VThf constant due to the volume guarantee (VG). VG is a well-documented volume target ventilation modality combined to synchronize conventional tidal ventilation [20], but this strategy has not been adequately evaluated during HFOV. 

To assess the efficacy of adjusting VThf settings instead of ΔPhf on PaCO_2_ and the stability of VThf and PaCO_2_ using VG ventilation during lung disease, an experimental study in a model of newborn piglets was conducted in which HFOV combined with VG was used before and after lung surfactant depression by bronchoalveolar lavage (BAL).

## 2. Materials and Methods

### 2.1. Animals

Six healthy 2-day-old Landrace-Large White piglets with a mean (±standard deviation, SD) body weight of 2.57 ± 0.26 kg were used. All animals were handled according to the Guide for the Care and Use of Laboratory Animals of the National Institutes of Health (http://grants.nih.gov/grants/olaw/Guide-for-the-Care-and-Use-of-Laboratory-Animals.pdf). Also, the ethics committee for animal care and use of the participating centers approved the study.

### 2.2. Anesthesia

General anesthesia was induced via facemask with 8% sevoflurane in oxygen. Then, a 24-gauge polyethylene catheter was placed into the ventrolateral auricular vein for continuous infusion of lactated Ringer's solution (10 mL/kg/h) and the administration of drugs. Once an adequate level of anesthesia was achieved, an endotracheal tube (3 mm inner diameter) was placed and secured. Anesthesia was maintained with an intravenous (i.v.) constant infusion of propofol as needed, and intraoperative analgesia was achieved with i.v. morphine chlorhydrate (1 mg/kg). 

### 2.3. Ventilation and Procedures

Animals were ventilated with HFOV combined with VG ventilation (Babylog VN500, Dräger, Lübeck, Germany). The following parameters were set: inspired oxygen fraction (FiO_2_) of 0.3; continuous distending pressure of the airway (mean airway pressure (mPaw) of 10 cm H_2_O; tidal volume on HFOV (VThf) set as VG modality of 2 mL/kg; and respiratory frequency (*f*_*R*_) of 10 Hz with a 1 : 1 inspiratory to expiratory ratio. The pressure swing around the mPaw (delta *P* [ΔPhf]) and the carbon dioxide diffusion coefficient (DCO_2_) calculated as *Vt* ^2^ × *f*_*R*_ (mL^2^/sec) were obtained from the ventilator software (VentView 2.n software, Dräger, Lübeck, Germany). 

Self-adhering parches were applied to the skin for ECG and heart rate (HR) recording. Pulse oximetry (SpO_2_) was recorded continuously by placing a pulse oximeter on the paw, and rectal temperature (*T* ^*a*^) was also monitored and maintained between 36°C and 38°C by means of a total temperature management system. By surgical cut down, the endotracheal tube was sealed with a ribbon wrapped around the trachea to prevent any leak, and 18-gauge polyethylene catheter was inserted into right carotid artery. This access allowed for arterial blood sampling and continuous blood pressure measurement via a calibrated pressure transducer. *T* ^*a*^, SpO_2_, HR, and arterial blood pressure (BP) (referred to a zero level) were registered continuously on a cardiovascular monitor (PM8060 Vitara, Dräger, Lübeck, Germany). Arterial blood was withdrawn anaerobically and immediately analyzed for pH, partial pressure of oxygen (PaO_2_), partial pressure of carbon dioxide (PaCO_2_), and arterial oxyhemoglobin saturation (SaO_2_) (IL 1306 pH/Blood Gas Analyzer, Allied Instrumentation).

Following instrumentation and after a stabilization period of 15 min, baseline cardiorespiratory parameters and arterial blood samples were collected at a VThf of 2 mL/kg. Then, VThf was increased to 2.5 and 3 mL/kg at 15 min intervals. After each change in VThf, a 15 min period of stabilization was allowed before recording parameters. This was followed by BAL with 3 aliquots of 10 mL/kg isotonic saline solution warmed to body temperature [21, 22] to induce surfactant depletion mimicking neonatal respiratory distress syndrome.

 To ensure adequate alveolar reexpansion, a sustained high-pressure recruitment maneuver using repeated 40 cm H_2_O of pressure during 30 s was undertaken following BAL. A period of 30 min was determined to establish the surfactant-depleted situation, while the ventilator was set on HFOV with a VG of 3 mL/kg, and then arterial blood tests were done.

Data from the ventilator were exported through the standard USB connection.

### 2.4. Statistical Analysis

Statistical analysis was performed using SPSS 20.0 software program (IBM SPSS Statistics, Chicago, IL, USA). Continuous data were analyzed using an analysis of variance (ANOVA) for repeated measurement with the Bonferroni's correction for multiple testing. Data are expressed as mean ± SD. Statistical significance was set at *P* < 0.05.

## 3. Results

There were no differences in HR, arterial BP, and *T* ^*a*^ among the animals during the study period (Table 1). The mean values of VThf for 2, 2.5, and 3 mL/kg were 5.0 ± 0.6, 6.2 ± 0.9, and 7.6 ± 0.9 mL, respectively. The FiO_2_ was maintained at 0.3 except during the BAL and the recruitment maneuvers (data not shown) when it was temporally increased as needed. Also, the frequency (10 Hz), mPaw (10 cm H_2_O), and the inspiratory to expiratory ratio (1 : 1) were maintained constant throughout the study.

Mean values for all the animals of pH, PaO_2_, PaCO_2_, Δ*P*, and DCO_2_ are shown in Table 2 at different VThf mL/kg during the physiological lung condition and after the BAL. HFOV with VG modality led to an increase in VThf, decrease in PaCO_2_, and a significant increase in DCO_2_ and measured ΔPhf (Table 2). 

**Figure 1:**
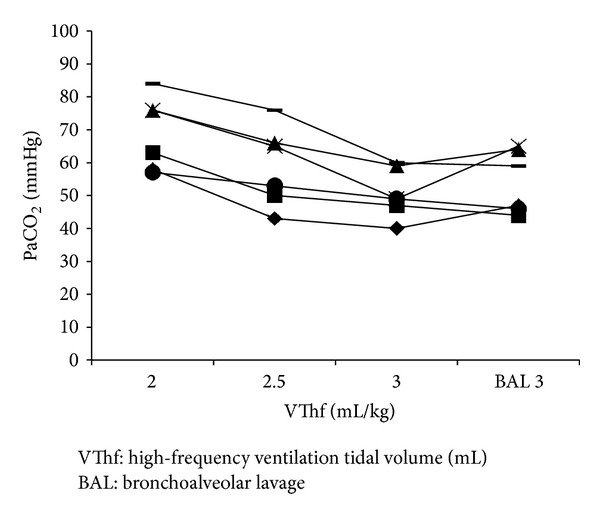
PaCO_2_ determined at different VThf before and after surfactant depletion by bronchoalveolar lavage (BAL).

**Figure 2:**
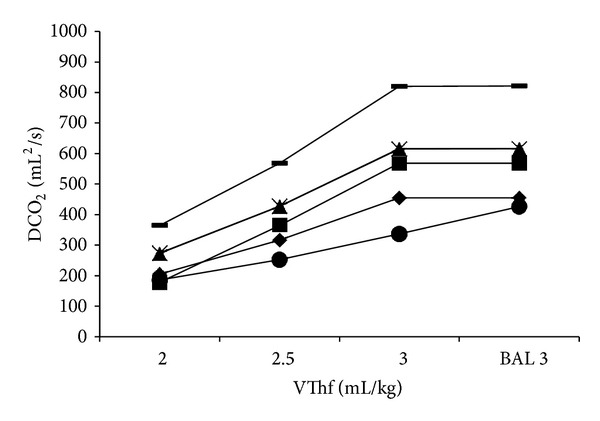
Carbon dioxide diffusion coefficient (DCO_2_) calculated at different VThf before and after surfactant depletion by bronchoalveolar lavage (BAL). DCO_2_: carbon dioxide diffusion coefficient calculated as *Vt* ^2^ × *f*_*R*_ (mL^2^/sec).

PaCO_2_ was inversely proportional to VThf in all animals and remained constant after surfactant depletion, as compared with the pre-BAL situation (Figure 1). DCO_2_ obtained from the ventilator increased with every increase in VThf during the physiological lung condition. After the BAL, DCO_2_ remained constant (Figure 2) in all animals.

**Figure 3:**
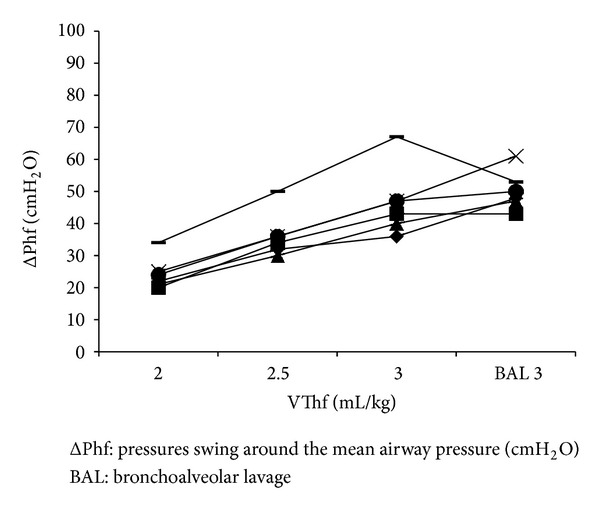
ΔPhf determined at different VThf before and after surfactant depletion by bronchoalveolar lavage (BAL).

Measured ΔPhf increased in all the animals as VThf was increased in the lung physiological condition; after the BAL, ΔPhf increased in three animals, remained unchanged in one, and decreased in two, as the VThf remained constant in all of them (Figure 3); as well as the PaCO_2_.

## 4. Discussion

In this study, using HFOV combined with VG in an animal model of neonatal distress syndrome during physiologic lung condition and after surfactant depletion, PaCO_2_ was dependent on VThf. For every increase in VThf, an increase in ΔPhf and a significant decrease in PaCO_2_ were observed. Using HFOV when the VG modality is not in use, fine-tuning changes of the ΔPhf and of the frequency of the ventilator have to be done to achieve the desired PaCO_2_. In that situation, when ΔPhf is increased, VThf increases and more CO_2_ is removed from the airways [6]. 

In our study, using HFOV combined with VG, for any increase in VThf there was a significant decrease in the PaCO_2_, which was accompanied by an increase in ΔPhf, as this was done by the ventilator in response to the increase in VThf settings. We used a VThf of 2 to 3 mL/kg, as the expected dead space has been thought to be about 2.2 mL/kg in healthy awake neonates [23, 24] and the median delivered volume has been documented to be 2.4 mL/kg (range 1.0 to 3.6) [16].

The exact mechanism for CO_2_ removal when there is an increase of VThf during HFOV combined with VG is not fully understood, as the tidal bulk flow cannot explain it alone, and the VThf generated is lower or similar to the anatomical dead space, so a combination of several mechanisms is probably involved [25]. Also, although tidal volume has a greater effect on CO_2_ elimination during HFOV than frequency [26], changes in the frequency induced changes in CO_2_ removal, but probably by an independent mechanism [8]. When the VG modality is not in use in HFOV, as frequency is decreased, PaCO_2_ decreases as well, and an increase in VThf may probably account for this finding [13], but some other mechanisms are probably involved, such as a better transmission of the pressure waves through the airways at lower frequencies [25]. It has been suggested that the optimal range of frequencies depends on both the body size and the intrinsic lung mechanics [27], and because finding this frequency is sometimes difficult, the use of a simple marker to guide settings for alveolar ventilation during HFOV will help on CO_2_ removal. During HFOV, changes in the frequency will also modify ΔPhf to both proximal and distal lung compartments. Using HFOV without the VG modality, it was recommended to set the highest frequency possible, while not compromising the VThf to prevent insufficient ventilation, to minimize ΔPhf, and to decrease barotrauma to the airways [15]. However, with the VG modality it is possible to combine the best frequency and modifying VThf, and this can be a preventive strategy to decrease lung damage.

We maintained a constant frequency as VThf was modified in order to assess better the isolated effect of the changes in VThf on PaCO_2_, and a close relationship was found between both. So this VG modality will be more efficient not only at maintaining a more stable PaCO_2_ but also to obtain fine modifications of the PaCO_2_ and a better monitoring system during HFOV. 

DCO_2_ calculated as *Vt* ^2^ × *f*_*R*_ (mL^2^/s) can be a marker of alveolar ventilation during HFOV. PaCO_2_ increases as DCO_2_ decreases, indicating alveolar hypoventilation [28]. In the present study, a clear effect of the changes of VThf on DCO_2_ was consistently observed in all animals during the physiological lung conditions. For any increase in VThf, there was a corresponding increase in DCO_2_. As the frequency was maintained unchanged, increases in DCO_2_ were only related to VThf increases. Using HFOV with VG, any increase in VThf produced a significant increase in DCO_2_ and a decrease in PaCO_2_.

Also, and probably because of the use of a constant VThf and frequency when using the VG modality, after the surfactant lung-depleted situation, no changes in DCO_2_ were found. In this respect, as may be expected, a constant and unchanged PaCO_2_ was observed.

When the ventilator is using the VG modality during HFOV, the VThf is maintained constant, so the ventilator modifies ΔPhf to prevent any change in VThf. After the surfactant deficiency situation, the ventilator modified ΔPhf in all the animals to maintain VThf constant, and probably this is the main reason why PaCO_2_ did not show a significant variation with any change in the lung condition. After the BAL, lung mechanics change fast, and an increase in ΔPhf was observed in three of the animals; in one there was no change, and in two there was a decrease. BAL produces a decrease in lung compliance [21, 22] that may affect VThf. 

The effect of surfactant depletion on lung mechanics is variable, and because of the recruiting strategy used to prevent lung atelectasis, continuous changes are expected during and after the first minutes of lung lavage, so the use of the VG modality maintained the VThf stable throughout the study. In three of the animals the ventilator increased ΔPhf to maintain VThf, no change was observed in one animal and a decrease in ΔPhf was found in two, showing how VThf was maintained constant by the ventilator in any lung mechanics condition. This would add an advantage in the management of respiratory problems in the presence of a sudden change in lung mechanics. The ventilator, using the VG modality, will automatically modify ΔPhf to maintain a constant VThf, as it was demonstrated in this study, and because of that, significant variations of the PaCO_2_ can probably be prevented. As it was expected, changes in lung mechanics produced by the surfactant depletion and the recruitment maneuvers were not uniform in all animals, mostly after the first minutes of the procedure, but as shown in the study, the ventilator using the VG modality maintains a constant VThf in any lung situation and in all the animals. After depletion of lung surfactant a low compliance situation is expected, but the effect of a decrease in the compliance on the VThf is variable. In has been demonstrated that modifications in respiratory compliance and mean airway pressure induced unexpected changes in VThf delivery, and also can vary depending on the type of ventilator. A decrease in VThf following an increase in lung compliance with the OHF 1 ventilator or an increase with the SensorMedics 3100^a^  has been reported [18]. High-frequency tidal volume is affected by the size of the endotracheal tube and patency of the airways, but is relatively insensitive to changes in lung tissue or chest wall mechanical properties [7]. This may partially explain why changes in Δ*P* were observed in only half of the animals after the BAL, as differing levels of compliance had little effect on delivered tidal volume related to the endotracheal tube size. The ventilatory frequency has an important impact on airway reopening [29], but the effect of varying lung compliance on delivered tidal volume may be explained by the expected behavior of a simple harmonic (or oscillating) system. Modifying the frequency below or above the resonant frequency of a system, increasing compliance lowers impedance, resulting in greater flows for the same pressure, or the inertial effects dominate impedance, and compliance has little effect on its value. If has been demonstrated that changes in test lung compliance had a negligible effect on delivered tidal volume [30].

This situation can occur in a newborn with a severe respiratory distress syndrome (RDS) as the lung mechanics can change and the VThf sent from the ventilator can change when ΔPhf instead of the VThf is set, so the use of the VG modality in those unstable conditions will probably prevent large variations in the PaCO_2_. The variables exhibiting the greatest effect on the magnitude of delivered VT are frequency, Δ*P*, and endotracheal tube internal diameter [30].

In relation to a decrease of VThf seen with some ventilators when test lung compliance increased, this was mainly due to a decrease in Δ*P* [18], so that using a VG strategy with an automatic adjust of Δ*P* by the ventilator probably will prevent such variations in VThf when lung compliance changes.

The present findings should be interpreted taking into account some limitations of the study. The animal model does not reproduce the clinical condition of a newborn with severe RDS before and after giving surfactant. Because the aim of the study was to assess the effect of modifying directly the VThf instead of Δ*P*, a physiological situation was designed firstly in order to show this effect better. No previous studies have been published using the HFOV combined with VG showing this clear effect on arterial PCO_2_ after an increase in the VThf during HFOV; therefore, a physiologic lung condition and the response of the PaCO_2_ after modifications in the VThf were designed. As the effect of BAL on lung mechanics of the animals was not homogeneous, we also assessed how the ventilator would maintain constant VThf modifying or not Δ*P* after BAL depending on the change of the lung mechanics. Also, the BAL effect on lung mechanics is variable among the animals as a recruitment strategy was used immediately after the lavage to prevent a severe atelectasis. 

## 5. Conclusions

The use of HFOV combined with VG ventilation in this newborn animal model of surfactant deficiency allowed for setting the VThf instead of ΔPhf to modify CO_2_ removal from the lung. A significant decrease in PaCO_2_ with any increase in VThf was found. For any change of the VThf setting, the ventilator modified ΔPhf to maintain the VThf. After BAL, the ventilator modified ΔPhf to maintain a constant VThf as needed, and PaCO_2_ remained unchanged compared to the pre-surfactant depletion condition. 

## Figures and Tables

**Table 1:** Physiological variables at different VThf before and after surfactant depletion by BAL.

VThf/kg	HR (beats per min)	Mean BP (mmHg)	*T* ^*a*^ (°C)
2 mL/kg	160 ± 24	53 ± 10	37.2 ± 0.6
2.5 mL/kg	160 ± 17	53 ± 10	37.1 ± 0.8
3 mL/kg	149 ± 15	54 ± 11	37.1 ± 0.8
BAL	NA	NA	NA
3 mL/kg	137 ± 19	47 ± 8	36.0 ± 0.7

NA: not available.

Data as mean ± SD.

**Table 2:** Physiological variables at different volumes guarantee before and after surfactant depletion by BAL.

VThf/kg	pH	PaO_2_ (mmHg)	PaCO_2_ (mmHg)	DCO_2_ (mL^2^/sec)	ΔPhf (cm H_2_O)
2 mL/kg	7.16 ± 0.064	78 ± 7	69 ± 11	247 ± 71	24 ± 6
2.5 mL/kg	7.22 ± 0.064	99 ± 11*	59 ± 12*	392 ± 109*	36 ± 8*
3 mL/kg	7.300 ± 0.074^∗,†^	107 ± 11*	51 ± 8*	569 ± 164^∗,†^	47 ± 12*
BAL	NA	NA	NA	NA	NA
3 mL/kg	7.24 ± 0.079	83 ± 14^†,‡^	54 ± 10*	584 ± 141^∗,†^	50 ± 7*

NA: not applicable.

Data as mean ± SD.

*P < 0.05 (ANOVA) as compared to 2 mL/kg.

^†^ *P* < 0.05 (ANOVA) as compared to 2.5 mL/kg.

^‡^ *P* < 0.05 (ANOVA) as compared to 3 mL/kg before BAL.

## List of Abbreviations

HFOV: High-frequency oscillatory ventilation
VThf: High-frequency expired tidal volume
CO_2_: Carbon dioxide
*f*_*R*_: Frequency
ΔPhf: Delta pressure
mPaw: Mean airway pressure
VG: Volume guarantee
BAL: Bronchoalveolar lavage
SD: Standard deviation
i.v.: Intravenous
FiO_2_: Inspired oxygen fraction
DCO_2_: Carbon dioxide diffusion coefficient
HR: Heart rate
SpO_2_: Pulse oximetry
*T*^*a*^: Rectal temperature
BP: Blood pressure
PaO_2_: Partial arterial oxygen pressure
PaCO_2_: Partial carbon dioxide pressure
SaO_2_: Arterial oxyhemoglobin saturation
RDS: Respiratory distress syndrome.
